# Evaluating change of function after revascularization in patients with multi vessel coronary artery disease, severely reduced left ventricular systolic function and no scar on CMR imaging

**DOI:** 10.1186/1532-429X-17-S1-P169

**Published:** 2015-02-03

**Authors:** Alexander Ivanov, James Yossef, Ambreen Mohamed, Joshua Socolow, Iossif Gulkarov, Berhane Worku, Pairoj Chattranukulchai, Anthony Tortolani, Terrence Sacchi, Mohamad G Ghosn, Dipan J Shah, John D Grizzard, Robert W Biederman, Igor Klem, John Heitner

**Affiliations:** Medicine, Division of Cardiology, New York Methodist Hospital, Brooklyn, NY USA; Cardiology, Duke Medical Center, Durham, NC USA; Cardiothoracic Surgery, New York Methodist Hospital, Brooklyn, NY USA; Houston Methodist Hospital, Houston, TX USA; Virginia Commonwealth University, Richmond, VA USA; Allegheny General Hospital, Pittsburgh, PA USA

## Background

Cardiac Magnetic Resonance (CMR) can accurately distinguish ischemic (ICM) versus non- ischemic cardiomyopathy (NICM) with very high sensitivity and specificity based on the presence and distribution of fibrosis. The absence of scar in patients with severely reduced left ventricular ejection fraction (LVEF) and non-obstructive coronary artery disease is very specific for NICM. Patients with 3-vessel coronary artery disease (CAD) amenable to coronary artery bypass grafting (CABG) and severely reduced LVEF who have no scar detected by CMR poses a potential clinical challenge. Improvement in myocardial function post-operatively in this subgroup of patients is largely unknown.

The aim of this study is to assess if this population of patients have improvement in LVEF after revascularization as compared to patients with scar and low LVEF.

## Methods

We assessed 64 patients from five centers, who had LVEF ≤35 % on CMR prior to CABG and underwent LVEF reassessment within 1 year post surgery. Patients were divided into 5 groups: 1. No scar (n = 6) 2. Viable myocardium (n = 43) 3. Non-viable myocardium (n = 9) 4. Non-ischemic scar (n = 3), 5. Combined ICM and NICM scar pattern (n = 3). We defined a non-viable group as the presence of 5 or more segments with ≥ 50% scar. The primary outcome was improvement in the LVEF post-CABG. A linear regression with categorical predictors was performed comparing Group 1 to the other Groups.

## Results

There was no difference in age, gender and baseline LVEF between groups. The mean LVEF increased 6 % in group 1, increased 10 % in group 2 (β= 4.6), increased 4% in group 3 (β= -2), improved 21% in group 4 (β= 14.9), and increased 11 % in group 5 (β= 4.9). There was no statistically significant difference between patients with no scar and the other groups. Sixty six percent of the patients with no scar had an LVEF improvement (mean 15%), and 81% of the patients from the rest of the groups had an LVEF improvement (mean 13%). Respectively, 33% of the patients with no scar had an LVEF decline (mean 13%) and 19% of the patients from the rest of the groups had an LVEF decline (mean 6%).

## Conclusions

Patients with 3-vessel CAD, severely reduced LVEF and no fibrosis detected by CMR are a heterogeneous population with differences in clinical outcome after revascularization. Further investigation into determining which patients might improve with revascularization is warranted.

## Funding

No extramural funding was used to support this work. The authors are solely responsible for the design and conduct of this study, all data analysis, drafting, editing of the abstract and its final content.

**Figure 1:**
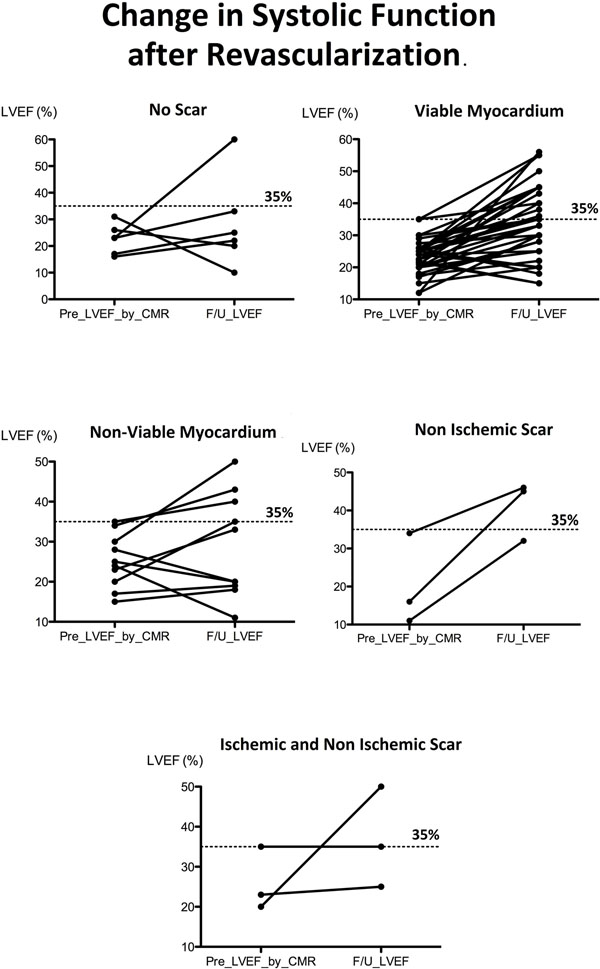
Figure 1

